# Prevalence and associated factors of paternal stress, anxiety, and depression symptoms in the early postnatal period

**DOI:** 10.1017/gmh.2022.33

**Published:** 2022-07-13

**Authors:** Lloyd Frank Philpott, Patricia Leahy-Warren, Serena FitzGerald, Eileen Savage

**Affiliations:** School of Nursing and Midwifery, Brookfield Health Sciences Complex, University College Cork, Cork T12AK54, Republic of Ireland

**Keywords:** Anxiety, depression, fatherhood, mental health, paternal, postnatal period, stress, symptoms

## Abstract

**Background:**

The changes experienced during the transition to first-time or subsequent fatherhood are mainly positive; however, fathers can also experience adverse mental health outcomes such as stress, anxiety, and depression. The aim of this study was to investigate the prevalence and associated factors of paternal stress, anxiety, and depression symptoms in the early postnatal period.

**Methods:**

A quantitative, descriptive correlational design was used. Data were collected using a self-administered questionnaire comprising of the Perceived Stress Scale, the State-Trait Anxiety Inventory, and the Edinburgh Postnatal Depression Scale.

**Results:**

A total of 336 fathers were included in the study. The prevalence rates were 41.1% (*n* = 138) for moderate/high stress symptoms, 20.8% (*n* = 70) for state anxiety symptoms, 25.9% (*n* = 87) for trait anxiety symptoms, and 13.4% (*n* = 45) for depression symptoms. In the multivariable analysis, several factors were associated with increased stress, anxiety, and depression symptoms including being a subsequent father (*p* = 0.009), not living in a house (*p* = 0.009), having a history of adverse mental health (*p* = 0.008), and having a partner with a history of anxiety (*p* = 0.040).

**Conclusion:**

The findings suggest that fathers are at risk of adverse mental health in the early postnatal period which is a pivotal time for fathers in terms of bonding with their infant and redefining their relationship with their partner.

## Background

The transition to first-time or subsequent fatherhood necessitates a change from a familiar existing reality to an unfamiliar new reality, where fathers need to balance family and work life, care for a new-born infant, and adjust to relationship changes with their partner (Genesoni and Tallandini, 2009; Glasser and Lerner-Geva, 2018). In general, the changes experienced during the transition are positive and lead to personal growth; however, fathers can also experience adverse mental health such as stress, anxiety, and depression (Cameron *et al*., 2016; Philpott *et al*., 2017, 2019). For the majority of fathers who experience adverse mental health, their responses are transient and adaptive in nature; however, for a small but significant number of fathers, non-adaptive symptomatology may contribute to the development of new or a re-emergence of mental health concerns and/or diagnosis (Pinto *et al*., 2016; Seah and Morawska, 2016). The most commonly researched adverse paternal mental health outcome during the early postnatal period is depression (Cameron *et al*., 2016); however, more recently, stress and anxiety have also begun to emerge as significant mental health concerns (Leach *et al*., 2016; Philpott *et al*., 2017).

### Stress symptoms

Lazarus and Folkman (1984) in their Transactional Model of Stress and Coping defined stress as ‘a particular relationship between the person and the environment that is appraised by the person as taxing or exceeding his or her resources and endangering his or her well-being’ (p. 19). During the early postnatal period fathers face many new stressors arising from their new-born's needs (Hildingsson and Thomas, 2014) and relationship changes (Kamalifard *et al*., 2014). To date one systematic review has investigated paternal stress and reported that fathers' stress symptoms increased from the antenatal period to the time of birth and early postnatal period, with a decrease in stress in the later postnatal period (Philpott *et al*., 2017). Based on screening tools, Philpott *et al*. (2017) in their systematic review reported a prevalence of stress symptoms among fathers during the perinatal period between 6% and 8.7%. Besides stress, other paternal mental health adverse outcomes that have been researched include anxiety.

### Anxiety symptoms

Spielberger (1972, p. 12) in his State–Trait Anxiety theory defined anxiety as ‘as an emotional response to stimulus perceived as dangerous’. In Spielberger's theory, anxiety facilitates the avoidance of danger and is a normal adaptive response; however, anxiety becomes maladaptive when it interferes with functioning, becomes overly frequent, severe, and persistent (Spielberger, 1972; Beesdo *et al*., 2009). During the early postnatal period fathers face many new anxieties arising from the need to balance family and work life (Koh *et al*., 2015), supporting their partner and caring for their infant (Mahmoodi *et al*., 2017). Similar to stress symptoms, a recent systematic review reported that fathers' anxiety symptoms increased from the antenatal period to the time of birth and early postnatal, with anxiety decreasing in the later postnatal period (Philpott *et al*., 2019). Based on screening tools, Philpott *et al*. (2019) in their systematic review reported that the prevalence rate for anxiety symptoms ranged between 3.4% and 25.0% during the antenatal period and between 2.4% and 51% during the postnatal period. While there is increasing research interest in paternal anxiety, the predominant focus has been on depression (Leach *et al*., 2016).

### Depression symptoms

Depression is ‘a state of low mood, with symptoms such as sadness, fatigue, loss of interest, and loss of appetite’ [World Health Organisation (WHO), 2017, p. 7]. While fathers can experience many of these depression symptoms during the perinatal period (Cameron *et al*., 2016), for the majority their symptoms are transient and are linked to stresses associated with the transition to fatherhood, or the arrival of subsequent children. To date, two systematic reviews have been undertaken to assess paternal depression symptoms (Paulson and Bazemore, 2010; Cameron *et al*., 2016). Based on screening tools, both Paulson and Bazemore (2010) and Cameron reported the 3–6 month postnatal period as having the highest prevalence estimate of depression; however, Cameron *et al*. (2016) reported a much lower prevalence rate of 13.0% compared to Paulson and Bazemore (2010) who reported a rate of 25.6%.

To date there has only been one Irish study that has assessed paternal perinatal mental health. Philpott and Corcoran (2018) assessed depression symptoms in 100 fathers up to 1 year postnatally and reported prevalence rates of 12% for major depression [Edinburgh Postnatal Depression Scale (EPDS) cut-off score of ⩾12) and 28% for minor depression (EPDS cut-off score of ⩾9]. Studies assessing paternal perinatal mental health have reported wide variations in reported prevalence rates. This wide variation may be attributed to diverse settings, sample size, recruitment strategies, inclusion and exclusion criteria, assessment timepoints, cut-off scores, the use of different measurement tools, and the cultural setting of the study. The sociocultural context of fatherhood can potentially have an impact on perinatal mental health. For example, in cultures where patriarchy is the dominant ideology, and emphasis is placed on the fathers' role as a ‘breadwinner’ and provider (Firouzan *et al*., 2019), it has been reported that fathers are more susceptible to stress and anxiety. For fathers in patriarchal societies, worry about family finances and the cost linked with having a baby can act as a catalyst for increased stress and anxiety (Genesoni and Tallandini, 2009, Darwin *et al*., 2017). While fathers in Ireland are expected to play an equal role in caring for their infant, similar to international studies, Irish research shows significant gender differences in involvement in care for children, with fathers spending significantly less time on childcare than mothers (Smyth and Russell, 2021).

This study was undertaken in the early postnatal period which is considered one of the most challenging times for fathers due to the need to balance the numerous demands placed on them (Genesoni and Tallandini, 2009; Glasser and Lerner-Geva, 2018). Furthermore, it is a significant time for fathers as they begin to build their bond with their infant and redefine their relationships with their partner and wider society. The importance of optimum adaptation and well-being during the early postnatal period is of paramount significance given the potential negative outcomes of poor adaptation and wellbeing for fathers, their partner, and infant including excessive fatigue, adverse maternal mental health, and a variety of psychological and developmental disturbances in later childhood (Fisher, 2017). Currently in the literature there are different timeframes used to define the early postnatal period ranging from immediately after birth to 30 days postnatally (World Health Organisation, 2010; Yuan *et al*., 2021). In this study the infants were 0 to 4 days old.

To the best of our knowledge, this is the first study to assess the prevalence and associated factors of stress, anxiety, and depression symptoms in the early postnatal period with data generated from the same population. The simultaneous analysis of associated variables more readily assesses fathers' real-world experience, where they are likely to encounter multiple factors associated with stress, anxiety, and depression. Given the lack of clarity around the prevalence and associated factors of paternal stress, anxiety, and depression symptoms in the early postnatal period this study is both timely and warranted.

## Methods

### Aim of the study

The aim of this study was to investigate the prevalence of and associated factors for paternal stress, anxiety, and depression symptoms in the early postnatal period.

### Research design

A quantitative, descriptive correlational design was used.

### Sampling and participants

Fathers regardless of parity, who were aged ⩾18 years, able to read and write English, and who were visiting their partner following the birth of their new-born infant at one large regional maternity hospital in the southern region of Ireland were invited to take part in the study. Fathers completed the paper-based questionnaire at the maternity hospital. Fathers whose infant had a diagnosis of a birth defect, were admitted to the neo-natal unit, or who were stillborn were excluded. Non-probability, convenience sampling was used to recruit fathers.

A pilot study (*n* = 20) was undertaken at the maternity hospital to ascertain the time that it took to complete the questionnaire, to ensure the clarity of the questions, and the effectiveness of the instructions (Parahoo, 2014). Fathers who completed the pilot study did not highlight any questions as unclear and none of them made any suggestions about removing any of the questions; therefore, no changes were made to the questionnaire following the pilot study. For the majority of fathers, it took around 10 min to complete; however, one father commented that the questionnaire took him over 20 min to complete. With regards to logistics and feasibility of collecting data, no major issues arose during the pilot study. The data from the pilot study were included in the final analysis.

### Measurement tools

The paper-based questionnaire consisted of two sections. The first section consisted of questions regarding fathers' demographic information, their own mental health and that of their partner and their pregnancy, birth and labour history and experience (see Table 1). The second section consisted of three screening instruments used to assess paternal stress, anxiety, and depression symptoms (Cohen *et al*., 1983; Spielberger *et al*., 1983; Cox *et al*., 1987).

**Table 1.** Demographic characteristics and self-reported mental health history, *n* = 336^a^

	*n*	(%)^a^
Age (years): mean (s.d.)	35.6	(5.8)
Nationality		
Irish	282	(83.9)
Non-Irish	54	(16.1)
Highest level of education		
Primary level	1	(0.3)
Secondary level	94	(28.0)
Third level/university	241	(71.7)
Current relationship status		
Married	238	(70.8)
Co-habiting	76	(22.6)
In a relationship but not co-habiting	19	(5.7)
Single	3	(0.9)
Current employment status		
Full-time employment	278	(82.7)
Self-employed	36	(10.7)
Unemployed	11	(3.3)
Type of accommodation		
A house	305	(90.7)
An apartment	25	(7.4)
Other	6	(1.9)
Own your own home		
Yes	216	(64.3)
No, privately renting	100	(29.7)
No, social housing	20	(6)
History of mental health issues		
Yes	45	(13.4)
No	291	(86.6)
Mental health problem experienced		
Anxiety	31	(9.2)
Stress	22	(6.5)
Depression	22	(6.5)
Other	3	(0.9)
Partner history of mental health issues		
Yes	52	(15.5)
No	284	(84.5)
Partner mental health issues		
Depression	34	(10.1)
Anxiety	26	(7.7)
Stress	14	(4.2)
Other	5	(1.5)
Smoker	56	(16.7)
Yes	280	(83.3)
No		
Drinks alcohol		
Yes	270	(80.3)
No	66	(19.7)
First-time father		
Yes	170	(50.5)
No	166	(49.5)
Number of other children^a^, *n* = 166		
1	92	(55.5)
2	47	(28.3)
3	17	(10.2)
4	9	(5.4)
5	1	(0.6)
Experience of labour and birth positive		
Yes	305	(90.7)
No	31	(9.3)
Previous experience of labour and birth positive^a^, *n* = 156		
Yes	120	(76.9)
No	36	(23.1)
Planned pregnancy		
Yes	276	(82.1)
No	60	(17.9)
Feelings about being a father (again)		
Happy	336	(100)
Sad	0	(0)
Intend taking paternity leave		
Yes	286	(85.1)
No	50	(14.9)

a For non-first-time fathers.

The Perceived Stress Scale (PSS) (Cohen *et al*., 1983) was used to measure stress symptoms. The 10 items on the scale are rated on a 5-point Likert scale ranging from ‘Never’ to ‘Very often’. Scores can range from 0 to 40 with higher scores indicating higher perceived stress. Scores were categorised into low perceived stress (0–13); moderate perceived stress (14–26), and high perceived stress (27–40) (Swaminathan *et al*., 2016). In this study the scale demonstrated high reliability with a Cronbach's alpha value of 0.85.

The State-Trait Anxiety Inventory (STAI) (Spielberger *et al*., 1983) was used to measure anxiety symptoms. The STAI consists of two distinctive scales. The Anxiety-State Scale (S-Anxiety) measures how people currently feel with the Anxiety-Trait Scale (T-Anxiety) measuring how people feel in general. Each scale consists of 20 items rated on a 4-point Likert scale. Scores on each scale can range from 20 to 80 with higher scores representing increased anxiety symptoms. A cut-off score of 40 (⩾40) was used for screening anxiety symptoms (Julian, 2011; Emons *et al*., 2019). In this study the STAI demonstrated excellent reliability with each scale having a Cronbach's alpha value of 0.93.

The EPDS (Cox *et al*., 1987) was used to measure depression. The 10 items on the scale are rated on a 4-point Likert scale. Scores range from 0 to 30 (Cox *et al*., 1987). A cut-off of 9 (⩾9) was used to identify fathers at risk of minor depression with a cut-off of 12 (⩾12) for major depression (Matthey *et al*., 2001; Massoudi *et al*., 2016). In this study the scale demonstrated high reliability with a Cronbach's alpha value of 0.86.

### Data analysis

A data-coding framework was developed prior to data collection, so that answers obtained from the questionnaire could be transferred into IBM SPSS Statistics (version 25.0, IBM Corp, Armonk, NY, USA). Returned questionnaires were reviewed for completeness. Following screening and cleaning of the data file, questionnaires were entered into IBM SPSS Statistics and statistical analysis was performed. Continuous variables were described using mean and standard deviation (s.d.) when normally distributed and median and interquartile range (IQR) when non-normally distributed. Categorical variables were described using frequencies and percentages. The majority of independent variables were classified as categorical data; however, there were continuous data including age, and how many children subsequent fathers had. Ninety-five per cent confidence intervals (95% CIs) for prevalence were calculated using a binomial distribution (Clopper–Pearson exact method). Univariable and multivariable logistic regression was used to investigate factors associated with PSS, SATI, and EPDS. In the multivariable analysis, forward stepwise regression was used to identify the best predictors (only variables that were significant in the multivariate analysis are reported in Tables 2–5). Unadjusted and adjusted odds ratios (ORs) and their corresponding 95% CIs are presented. All tests were two-sided and a *p* value < 0.05 was considered statistically significant. Missing scores for individual items on the scales were replaced with the mean score of the non-missing items within the scale if at least 80% of the items had been answered.

### Ethical approval

Ethical approval was obtained from the Clinical Research Ethics Committee of the Cork Teaching Hospitals (CREC) prior to commencing the study.

## Findings

### Demographic characteristics and mental health history

In the study, fathers' infants ranged from 0 to 4 days. The majority of fathers in the study were Irish (*n* = 282, 83.9%), educated to third/university level (*n* = 241, 71.7%), married (*n* = 238, 70.8%), and in full-time employment (*n* = 278, 82.7%). The age of fathers ranged from 19 to 65 years with a mean (s.d.) of 35.6 (5.8) years. Just over half (*n* = 170, 50.5%) of the fathers were first-time fathers. Forty-five fathers (13.4%) self-reported having a history of adverse mental health including anxiety (*n* = 31, 9.2%), stress (*n* = 22, 6.5%), depression (*n* = 22, 6.5%), obsessive compulsive disorder (OCD) (*n* = 1), bipolar disorder (*n* = 1), and not specified (*n* = 1). Over 15% (*n* = 52, 15.4%) of respondents had a partner with a history of mental health problems. Depression was the most common mental health problem experienced (*n* = 35, 10.4%) followed by anxiety (*n* = 27, 8%) and stress (*n* = 15, 4.4%). Five respondents stated that their partners had a different mental health problem including OCD (*n* = 2), postnatal depression (*n* = 1), MS (*n* = 1), and not specified (*n* = 1). There were 14 couples (4.1%) where both the father and his partner had a history of adverse mental health outcomes (see Table 1).

### Stress symptoms

The prevalence was 58.9% (95% CI 53.5–64.2) for low stress symptoms, 39.9% (95% CI 34.6–45.3) for moderate stress symptoms, and 1.2% (0.3%–3.0%) for high stress symptoms. Based on the multivariable analyses, factors statistically significant for moderate/high stress symptoms were having an adverse mental health history (*p* = 0.008), a trait anxiety sore ⩾40 (*p* < 0.001), an EPDS score ⩾9 (*p* < 0.001), as well as not living in a house (*p* = 0.009) and being a subsequent father (*p* = 0.009) (see Table 2).

**Table 2.** Univariable and multivariable analyses to investigate relationships between fathers' characteristics and perceived stress, *n* = 336

Dependent variable: 1 = Moderate/high perceived stress 0 = Low perceived stress	Low perceived stress (*n* = 198)		Moderate/high perceived stress (*n* = 138)		Univariable analysis		Multivariable analysis	
	%	(*n*)	%	(*n*)	OR (95% CI)	*p* value	OR (95% CI)	*p* value
Third level education						0.221		
No	53.7	(51)	46.3	(44)	1			
Yes	61.0	(147)	39.0	(94)	0.74 (0.46–1.20)			
Relationship status						0.082		
In relationship but not cohabiting/single	40.9	(9)	59.1	(13)	1			
Married/cohabiting	60.2	(189)	39.8	(125)	0.46 (0.19–1.10)			
Employment status						0.060		
Full-time employment	61.9	(172)	38.1	(106)	1			
Self-employed	44.4	(16)	55.6	(20)	2.03 (1.01–4.09)			
Other	45.5	(10)	54.5	(12)	1.95 (0.81–4.66)			
Living in a house						0.030		0.009
No	40.6	(13)	59.4	(19)	1		1	
Yes	60.9	(185)	39.1	(119)	0.44 (0.21–0.92)		0.31 (0.13–0.75)	
Home owner						0.469		
No	56.3	(67)	43.7	(52)	1			
Yes	60.4	(131)	39.6	(86)	0.85 (0.54–1.33)			
Smoker						0.076		
No	61.1	(171)	38.9	(109)	1			
Yes	48.2	(27)	51.8	(29)	1.69 (0.95–3.00)			
Drinks alcohol weekly						0.387		
No	55.1	(49)	44.9	(40)	1			
Yes	60.3	(149)	39.7	(98)	0.81 (0.49–1.31)			
First-time father						0.051		0.009
No	53.6	(89)	46.4	(77)	1		1	
Yes	64.1	(109)	35.9	(61)	0.65 (0.42–1.00)		0.47 (0.26–0.83)	
Experience of labour and birth positive						<0.001		
No	28.1	(9)	71.9	(23)	1			
Yes	62.2	(189)	37.8	(115)	0.24 (0.11–0.53)			
Planned pregnancy						0.785		
No	57.4	(35)	42.6	(26)	1			
Yes	59.3	(163)	40.7	(112)	0.92 (0.53–1.62)			
Intend taking paternity leave						0.885		
No	58.0	(29)	42.0	(21)	1			
Yes	59.1	(169)	40.9	(117)	0.96 (0.52–1.76)			
Father has history of mental health problems						<0.001		0.008
No	64.6	(188)	35.4	(103)	1		1	
Yes	22.2	(10)	77.8	(35)	6.39 (3.04–13.43)		3.56 (1.40–9.05)	
Father has history of stress						<0.001		
No	61.8	(194)	38.2	(120)	1			
Yes	18.2	(4)	81.8	(18)	7.28 (2.40–22.01)			
Father has history of anxiety						<0.001		
No	62.6	(191)	37.4	(114)	1			
Yes	22.6	(7)	77.4	(24)	5.74 (2.40–13.76)			
Father has history of depression						0.001		
No	61.5	(193)	38.5	(121)	1			
Yes	22.7	(5)	77.3	(17)	5.42 (1.95–15.08)			
Partner has history of mental health problems						0.157		
No	60.6	(172)	39.4	(112)	1			
Yes	50.0	(26)	50.0	(26)	1.54 (0.85–2.78)			
Partner has history of stress						0.082		
No	59.9	(193)	40.1	(129)	1			
Yes	35.7	(5)	64.3	(9)	2.69 (0.88–8.22)			
Partner has history of anxiety						0.584		
No	59.4	(184)	40.6	(126)	1			
Yes	53.8	(14)	46.2	(12)	1.25 (0.56–2.80)			
Partner has history of depression						0.068		
No	60.6	(183)	39.4	(119)	1			
Yes	44.1	(15)	55.9	(19)	1.95 (0.95–3.98)			
High anxiety-state						<0.001		
No	69.9	(186)	30.1	(80)	1			
Yes	17.1	(12)	82.9	(58)	11.24 (5.72–22.06)			
High anxiety-trait						<0.001		<0.001
No	74.7	(186)	25.3	(63)	1		1	
Yes	13.8	(12)	86.2	(75)	18.45 (9.41–36.17)		8.71 (3.88–19.55)	
Depressive symptoms						<0.001		<0.001
No	73.0	(187)	27.0	(69)	1		1	
Yes – minor depressive symptoms	25.7	(9)	74.3	(26)	7.83 (3.49–17.54)		2.84 (1.08–7.50)	
Yes – major depressive symptoms	4.4	(2)	95.6	(43)	58.27 (13.74–247.02)		14.79 (3.10–70.64)	
Age					0.99 (0.96–1.03)	0.728		

### State anxiety symptoms

The prevalence of state anxiety symptoms was 20.8% (95% CI 16.6–25.6). In the multivariable analysis factors statistically significant for a high state anxiety score ⩾40 included being single/not cohabiting (*p* = 0.022), a high trait anxiety score ⩾40 (*p* < 0.001), and a moderate/high stress score ⩾14 (*p* = 0.042) (see Table 3).

**Table 3.** Univariable and multivariable analyses to investigate relationships between fathers' characteristics and anxiety-state, *n* = 336

Dependent variable: 1 = High anxiety-state 0 = Low anxiety-state	Low anxiety-state (*n* = 266)		High anxiety-state (*n* = 70)		Univariable analysis		Multivariable analysis	
	%	(*n*)	%	(*n*)	OR (95% CI)	*p* value	OR (95% CI)	*p* value
Third level education						0.033		
No	71.6	(68)	28.4	(27)	1			
Yes	82.2	(198)	17.8	(43)	0.55 (0.31–0.95)			
Relationship status						0.021		0.022
In relationship but not cohabiting/single	59.1	(13)	40.9	(9)	1		1	
Married/cohabiting	80.6	(253)	19.4	(61)	0.35 (0.14–0.85)		0.22 (0.06–0.80)	
Employment status						0.530		
Full-time employment	80.2	(223)	19.8	(55)	1			
Self-employed	72.2	(26)	27.8	(10)	1.56 (0.71–3.42)			
Other	77.3	(17)	22.7	(5)	1.19 (0.42–3.37)			
Living in a house						0.289		
No	71.9	(23)	28.1	(9)	1			
Yes	79.9	(243)	20.1	(61)	0.64 (0.28–1.46)			
Home owner						0.083		
No	73.9	(88)	26.1	(31)	1			
Yes	82.0	(178)	18.0	(39)	0.62 (0.36–1.06)			
Smoker						0.121		
No	80.7	(226)	19.3	(54)	1			
Yes	71.4	(40)	28.6	(16)	1.67 (0.87–3.21)			
Drinks alcohol weekly						0.098		
No	73.0	(65)	27.0	(24)	1			
Yes	81.4	(201)	18.6	(46)	0.62 (0.35–1.09)			
First-time father						0.516		
No	77.7	(129)	22.3	(37)	1			
Yes	80.6	(137)	19.4	(33)	0.84 (0.50–1.42)			
Experience of labour and birth positive						<0.001		
No	53.1	(17)	46.9	(15)	1			
Yes	81.9	(249)	18.1	(55)	0.25 (0.12–0.53)			
Planned pregnancy						0.919		
No	78.7	(48)	21.3	(13)	1			
Yes	79.3	(218)	20.7	(57)	0.97 (0.49–1.90)			
Intend taking paternity leave						0.875		
No	80.0	(40)	20.0	(10)	1			
Yes	79.0	(226)	21.0	(60)	1.06 (0.50–2.25)			
Father has history of mental health problems						0.003		
No	81.8	(238)	18.2	(53)	1			
Yes	62.2	(28)	37.8	(17)	2.73 (1.39–5.34)			
Father has history of stress						0.005		
No	80.9	(254)	19.1	(60)	1			
Yes	54.5	(12)	45.5	(10)	3.53 (1.46–8.55)			
Father has history of anxiety						0.001		
No	81.6	(249)	18.4	(56)	1			
Yes	54.8	(17)	45.2	(14)	3.66 (1.70–7.86)			
Father has history of depression						0.195		
No	79.9	(251)	20.1	(63)	1			
Yes	68.2	(15)	31.8	(7)	1.86 (0.73–4.75)			
Partner has history of mental health problems						0.422		
No	79.9	(227)	20.1	(57)	1			
Yes	75.0	(39)	25.0	(13)	1.33 (0.66–2.65)			
Partner has history of stress						0.171		
No	79.8	(257)	20.2	(65)	1			
Yes	64.3	(9)	35.7	(5)	2.20 (0.71–6.78)			
Partner has history of anxiety						0.428		
No	79.7	(247)	20.3	(63)	1			
Yes	73.1	(19)	26.9	(7)	1.44 (0.58–3.59)			
Partner has history of depression						0.683		
No	79.5	(240)	20.5	(62)	1			
Yes	76.5	(26)	23.5	(8)	1.19 (0.51–2.76)			
High anxiety-trait						<0.001		<0.001
No	95.2	(237)	4.8	(12)	1		1	
Yes	33.3	(29)	66.7	(58)	39.50 (19.01–82.09)		27.79 (11.83–65.27)	
Moderate/high perceived stress						<0.001		0.042
No	93.9	(186)	6.1	(12)	1		1	
Yes	58.0	(80)	42.0	(58)	11.24 (5.72–22.06)		2.44 (1.03–5.78)	
Depressive symptoms						<0.001		
No	91.4	(234)	8.6	(22)	1			
Yes – minor depressive symptoms	45.7	(16)	54.3	(19)	12.63 (5.70–27.99)			
Yes – major depressive symptoms	35.6	(16)	64.4	(29)	19.28 (9.10–40.84)			
Age					0.96 (0.91–1.01)	0.089		

### Trait anxiety symptoms

The prevalence of trait anxiety symptoms was 25.9% (95% CI 21.3–30.9). The statistically significant factors for a high trait anxiety score ⩾40 in the multivariable analysis were being a first-time father (*p* = 0.010), being married/cohabiting (*p* = 0.043), a high state anxiety score ⩾40 (*p* < 0.001), a moderate/high stress score ⩾14 (*p* < 0.001), and an EPDS score ⩾9 (*p* < 0.001) (see Table 4).

**Table 4.** Univariable and multivariable analyses to investigate relationships between fathers' characteristics and anxiety-trait, *n* = 336

Dependent variable: 1 = High anxiety-trait 0 = Low anxiety-trait	Low anxiety-trait (*n* = 249)		High anxiety-trait (*n* = 87)		Univariable analysis		Multivariable analysis		
	%	(*n*)	%	(*n*)	OR (95% CI)	*p* value	OR	(95% CI)	*p* value
Third level education						0.225			
No	69.5	(66)	30.5	(29)	1				
Yes	75.9	(183)	24.1	(58)	0.72 (0.43–1.22)				
Relationship status						0.513			0.043
In relationship but not cohabiting/single	68.2	(15)	31.8	(7)	1		1		
Married/cohabiting	74.5	(234)	25.5	(80)	0.73 (0.29–1.86)		6.30 (1.06–37.32)		
Employment status						0.615			
Full-time employment	75.2	(209)	24.8	(69)	1				
Self-employed	69.4	(25)	30.6	(11)	1.33 (0.62–2.85)				
Other	68.2	(15)	31.8	(7)	1.41 (0.55–3.61)				
Living in a house						0.904			
No	75.0	(24)	25.0	(8)	1				
Yes	74.0	(225)	26.0	(79)	1.05 (0.45–2.44)				
Home owner						0.276			
No	70.6	(84)	29.4	(35)	1				
Yes	76.0	(165)	24.0	(52)	0.76 (0.46–1.25)				
Smoker						0.014			
No	76.8	(215)	23.2	(65)	1				
Yes	60.7	(34)	39.3	(22)	2.14 (1.17–3.91)				
Drinks alcohol weekly						0.094			
No	67.4	(60)	32.6	(29)	1				
Yes	76.5	(189)	23.5	(58)	0.63 (0.37–1.08)				
First-time father						0.622			0.010
No	75.3	(125)	24.7	(41)	1		1		
Yes	72.9	(124)	27.1	(46)	1.13 (0.69–1.84)		3.31 (1.33–8.26)		
Experience of labour and birth positive						0.002			
No	50.0	(16)	50.0	(16)	1				
Yes	76.6	(233)	23.4	(71)	0.30 (0.15–0.64)				
Planned pregnancy						0.302			
No	68.9	(42)	31.1	(19)	1				
Yes	75.3	(207)	24.7	(68)	0.73 (0.40–1.33)				
Intend taking paternity leave						0.305			
No	80.0	(40)	20.0	(10)	1				
Yes	73.1	(209)	26.9	(77)	1.47 (0.70–3.09)				
Father has history of mental health problems						<0.001			
No	78.7	(229)	21.3	(62)	1				
Yes	44.4	(20)	55.6	(25)	4.62 (2.41–8.86)				
Father has history of stress						0.001			
No	76.4	(240)	23.6	(74)	1				
Yes	40.9	(9)	59.1	(13)	4.68 (1.93–11.40)				
Father has history of anxiety						<0.001			
No	77.0	(235)	23.0	(70)	1				
Yes	45.2	(14)	54.8	(17)	4.08 (1.91–8.68)				
Father has history of depression						0.011			
No	75.8	(238)	24.2	(76)	1				
Yes	50.0	(11)	50.0	(11)	3.13 (1.31–7.51)				
Partner has history of mental health problems						0.225			
No	75.4	(214)	24.6	(70)	1				
Yes	67.3	(35)	32.7	(17)	1.48 (0.78–2.81)				
Partner has history of stress						0.148			
No	74.8	(241)	25.2	(81)	1				
Yes	57.1	(8)	42.9	(6)	2.23 (0.75–6.62)				
Partner has history of anxiety						0.555			
No	74.5	(231)	25.5	(79)	1				
Yes	69.2	(18)	30.8	(8)	1.30 (0.54–3.11)				
Partner has history of depression						0.190			
No	75.2	(227)	24.8	(75)	1				
Yes	64.7	(22)	35.3	(12)	1.65 (0.78–3.50)				
High anxiety-state						<0.001			<0.001
No	89.1	(237)	10.9	(29)	1		1		
Yes	17.1	(12)	82.9	(58)	39.50 (19.01–82.09)		23.73 (8.84–63.72)		
Moderate/high perceived stress						<0.001			<0.001
No	93.9	(186)	6.1	(12)	1		1		
Yes	45.7	(63)	54.3	(75)	18.45 (9.41–36.17)		6.94 (2.75–17.49)		
Depressive symptoms						<0.001			<0.001
No	90.6	(232)	9.4	(24)	1		1		
Yes – minor depressive symptoms	31.4	(11)	68.6	(24)	21.09 (9.21–48.28)		9.03 (3.07–26.52)		
Yes – major depressive symptoms	13.3	(6)	86.7	(39)	62.83 (24.14–163.57)		20.70 (6.02–71.22)		
Age					0.96 (0.92–1.01)	0.093			

### Depression symptoms

The prevalence of minor or major depressive symptoms (EPDS score ⩾9) was 23.8% (95% CI 19.4–28.7) and the prevalence of major depressive symptoms (EPDS score ⩾12) was 13.4% (95% CI 9.9–17.5). In the multivariable analysis, having a history of adverse mental health (*p* = 0.025), a partner with a history of anxiety (*p* = 0.040), a high trait anxiety score ⩾40 (*p* < 0.001), and a moderate/high stress score ⩾14 (*p* = 0.002) were statistically significant for an EPDS score ⩾12 (see Table 5).

**Table 5.** Univariable and multivariable analyses to investigate relationships between fathers' characteristics and major depressive symptoms, *n* = 336

	Major depressive symptoms							
Dependent variable: 1 = Major depressive symptoms (score ⩾ 12) 0 = Major depressive symptoms (score < 12)	No (*n* = 291)		Yes (*n* = 45)		Univariable analysis		Multivariable analysis	
	%	(*n*)	%	(*n*)	OR (95% CI)	*p* value	OR (95% CI)	*p* value
Third level education						0.246		
No	83.2	(79)	16.8	(16)	1			
Yes	88.0	(212)	12.0	(29)	0.68 (0.35–1.31)			
Relationship status						0.056		
In relationship but not cohabiting/single	72.7	(16)	27.3	(6)	1			
Married/cohabiting	87.6	(275)	12.4	(39)	0.38 (0.14–1.02)			
Employment status						0.197		
Full-time employment	88.1	(245)	11.9	(33)	1			
Self-employed	80.6	(29)	19.4	(7)	1.79 (0.73–4.42)			
Other	77.3	(17)	22.7	(5)	2.18 (0.76–6.31)			
Living in a house						0.697		
No	84.4	(27)	15.6	(5)	1			
Yes	86.8	(264)	13.2	(40)	0.82 (0.30–2.25)			
Home owner						0.490		
No	84.9	(101)	15.1	(18)	1			
Yes	87.6	(190)	12.4	(27)	0.80 (0.42–1.52)			
Smoker						0.520		
No	87.1	(244)	12.9	(36)	1			
Yes	83.9	(47)	16.1	(9)	1.30 (0.59–2.87)			
Drinks alcohol weekly						0.266		
No	83.1	(74)	16.9	(15)	1			
Yes	87.9	(217)	12.1	(30)	0.68 (0.35–1.34)			
First-time father						0.571		
No	85.5	(142)	14.5	(24)	1			
Yes	87.6	(149)	12.4	(21)	0.83 (0.44–1.56)			
Experience of labour and birth positive						0.001		
No	65.6	(21)	34.4	(11)	1			
Yes	88.8	(270)	11.2	(34)	0.24 (0.11–0.54)			
Planned pregnancy						0.448		
No	83.6	(51)	16.4	(10)	1			
Yes	87.3	(240)	12.7	(35)	0.74 (0.35–1.60)			
Intend taking paternity leave						0.754		
No	88.0	(44)	12.0	(6)	1			
Yes	86.4	(247)	13.6	(39)	1.16 (0.46–2.90)			
Father has history of adverse mental health outcomes						<0.001		0.025
No	91.1	(265)	8.9	(26)	1		1	
Yes	57.8	(26)	42.2	(19)	7.45 (3.64–15.24)		2.81 (1.14–6.92)	
Father has history of stress						<0.001		
No	89.2	(280)	10.8	(34)	1			
Yes	50.0	(11)	50.0	(11)	8.24 (3.32–20.43)			
Father has history of anxiety						0.002		
No	88.5	(270)	11.5	(35)	1			
Yes	67.7	(21)	32.3	(10)	3.67 (1.60–8.44)			
Father has history of depression						<0.001		
No	88.9	(279)	11.1	(35)	1			
Yes	54.5	(12)	45.5	(10)	6.64 (2.67–16.50)			
Partner has history of adverse mental health outcomes						0.078		
No	88.0	(250)	12.0	(34)	1			
Yes	78.8	(41)	21.2	(11)	1.97 (0.93–4.20)			
Partner has history of stress						0.019		
No	87.6	(282)	12.4	(40)	1			
Yes	64.3	(9)	35.7	(5)	3.92 (1.25–12.27)			
Partner has history of anxiety						0.041		0.040
No	87.7	(272)	12.3	(38)	1		1	
Yes	73.1	(19)	26.9	(7)	2.64 (1.04–6.69)		4.18 (1.07–16.39)	
Partner has history of depression						0.199		
No	87.4	(264)	12.6	(38)	1			
Yes	79.4	(27)	20.6	(7)	1.80 (0.73–4.42)			
High anxiety-state						<0.001		
No	94.0	(250)	6.0	(16)	1			
Yes	58.6	(41)	41.4	(29)	11.05 (5.52–22.12)			
High anxiety-trait						<0.001		<0.001
No	97.6	(243)	2.4	(6)	1		1	
Yes	55.2	(48)	44.8	(39)	32.91 (13.20–82.04)		12.90 (4.65–35.76)	
Moderate/high perceived stress						<0.001		0.002
No	99.0	(196)	1.0	(2)	1		1	
Yes	68.8	(95)	31.2	(43)	44.36 (10.52–186.99)		11.96 (2.51–57.06)	
Age					0.95 (0.89–1.00)	0.061		

## Discussion

The aim of this study was to investigate paternal stress, anxiety, and depression symptoms in the early postnatal period. The average age of fathers in the study was 36 years which is similar to the national average age of new fathers in Ireland which is estimated at 35 years [Central Statistics Office (CSO), 2017]. The trends over the past decade in Ireland and across the developed world suggest that the age of new fathers is increasing (CSO, 2017; Halvaei *et al*., 2020). Changing patterns of education, employment, postponement of marriage, increased life expectancy, and improved success with assisted reproductive technologies have been contributing to increased paternal age (Halvaei *et al*., 2020). The fathers in the study were highly educated with nearly three quarters educated to third level. The national average for third level education in Ireland is just under half; however, nearly two-thirds of men aged 18–40 years possess a third level qualification (CSO, 2017). The findings suggest that the fathers in our study in terms of age, nationality, education, and employment are similar to the national average for new fathers (CSO, 2017).

In this study, stress was common with just under half of the fathers having moderate/high symptoms. Paternal stress in the early postnatal period is an important indicator of well-being, as excessive stress demonstrates evidence that parenting demands exceed one's ability to comfortably meet those demands (Koester and Petts, 2017). Increased stress during this period highlights the demands of adjusting to early parenting (specifically, those associated with sleep and feeding) and altered relationships (Darwin *et al*., 2017). The early postnatal period is relatively soon after the stressful life events of labour and childbirth (McKelvin *et al*., 2020) and is marked by much change, the absence of routine, and the need to balance many responsibilities (Shorey *et al*., 2017). All these factors, have the potential to increase stress (Shorey *et al*., 2018), which can impact negatively on a fathers' ability to parent effectively and on his relationship with his partner during this critical time in the early postnatal period (Kamalifard *et al*., 2014, Bakermans-Kranenbur *et al*., 2019).

Trait anxiety was more common than state anxiety. According to Spielberger (1972), individuals with high levels of trait anxiety are more susceptible to stress and respond to situations as if they are dangerous or threatening. Furthermore, individuals with high levels of trait anxiety show state anxiety reactions more frequently and with greater intensity than those with low trait anxiety. Perhaps this should be expected as labour and birth have been reported as stressful life events by fathers (Darwin *et al*., 2017), and the early postnatal period is marked by many potential stressors such as the need to balance numerous demands including personal and work commitments, economic pressures, and fatigue (Genesoni and Tallandini, 2009; Glasser and Lerner-Geva, 2018). The link between stressful life events and the origin and development of anxiety has been investigated in populations outside the perinatal period and provides evidence for supporting the association (Gold, 2015; Phillips *et al*., 2015; Zuo *et al*., 2020). Indeed, as fathers perceive labour, childbirth, and the early postnatal period as stressful, the potential risk for anxiety is increased (Etheridge and Slade, 2017; Philpott *et al*., 2017).

A considerable number of fathers had an elevated EPDS score. Indeed, the true prevalence of depressive symptoms may be even greater given that masculine gender norms where depression is perceived as emotional weakness leads to under reporting and under detection (O'Brien *et al*., 2017). Additionally, while men and women express depression as low mood with reduced activity, depression in men is often masked with externalising behaviours and disorders such as substance abuse, avoidance behaviour, and anger (Chhabra *et al*., 2020). Masked depression, resulting in externalising symptoms such as physical illness, alcohol and drug abuse, and domestic violence, is likely to lead to under detection (Marcus *et al*., 2012; Chhabra *et al*., 2020).

The study also investigated statistically significant factors for stress, anxiety, and depression symptoms. Two factors were unique for increased stress symptoms in this study. The first unique factor was being a subsequent father. This factor is interesting as much of the research to date has focused on the challenges experienced by first-time fathers (Márquez *et al*., 2019; Ngai and Lam, 2020). The results reported from previous studies relating to increased stress symptoms for subsequent fathers are inconclusive (Hildingsson *et al*., 2014; Wee *et al*., 2015). Despite the inconclusive findings, it is suggested that past experiences can provoke a paternal stress reaction due to a previous negative birth experience, caring for an unsettled infant or difficulties in repositioning themselves in relation to their partner, child, and work (Darwin *et al*., 2017; Shorey and Chan, 2020). Subsequent fathers also have the added stress of caring for more than one child (Darwin *et al*., 2017). Although subsequent fathers can gain reassurance from knowing that many of the challenges that they face are transient, the demands of meeting the needs of more than one child can trigger a stress response and increase stress symptoms. It has been reported that there is an expectation that subsequent fathers are better prepared by virtue of their previous experiences and as a result they are often overlooked in clinical practice (Shorey *et al*., 2017). The findings of this study suggest that the lack of focus on subsequent fathers is an oversight; however, it is widely reported that the mental health of both first-time and subsequent fathers is generally overlooked, and their needs are often unmet (Baldwin *et al*., 2019) and there are currently no specialised supports provided by the government in Ireland for fathers experiencing adverse mental health during the perinatal period (Health Service Executive, 2017).

The second unique factor for increased stress symptoms was not living in a house. While apartment living has been standard in most mainland European cities and many other countries across the globe, Irish culture has continually associated apartments with the first step on the property ladder or a downsize equity in later years. As a consequence, it has been suggested that there is a significant shortage of apartments suited to family occupation in Ireland (Mooney, 2016). In Ireland, apartments only account for 12% of all households (Central Statistics Office, 2016). The majority of fathers in this study who did not live in a house, lived in an apartment, with only six fathers living in another type of accommodation. This factor is a new finding as it has not been reported in previous studies that assessed paternal stress. It is also an important finding as it is during this critical time in the early postnatal period when fathers can become aware of the restricted living space and lack of recreational spaces associated with apartment living (Kopec, 2006; Barton and Rogerson, 2017). It is suggested that reduced space associated with apartment living affects the overall well-being and comfort of the occupants (Kopec, 2006; Hu and Coulter, 2017) and can lead to living dissatisfaction, discomfort, and adverse mental health (Hu and Coulter, 2017). The relevance of this finding is highlighted by the fact that cities across the world are becoming more densified which will result in more people living in apartments with reduced space (Griffiths *et al*., 2017). Therefore, addressing the impact that such living spaces have on paternal, and family mental health well-being becomes more urgent and crucial.

A statistically significant factor for trait anxiety was being married/cohabiting, while a statistically significant factor for state anxiety was being single/not cohabiting. These findings are important as they seem to highlight that as well as a fathers' relationship status, the quality of his relationships and the support network associated with such relationships may play an important role in determining his mental health (Gao *et al*., 2009; Mao *et al*., 2011; Koh *et al*., 2015; Chhabra *et al*., 2020). Previous research has tended to focus on relationship status rather than on relationship quality (Chhabra *et al*., 2020), with marriage been identified as a protective factor; however, in a small number of studies, relationship quality and marital distress have been reported as risk factors for adverse mental health (Bergström, 2013; Koh *et al*., 2015; Nishimura *et al*., 2015). Distress in a marital relationship can diminish partner support and is likely to add to the anxiety related to the birth of an infant (Bergström, 2013; Nishimura *et al*., 2015). Previous research has highlighted that single fathers experience diminished co-parenting support and social support which have been identified as a risk factor for adverse mental health in the postnatal period (Gao *et al*., 2009; Mao *et al*., 2011; Koh *et al*., 2015).

Many statistically significant factors for depressive symptoms were identified and included a self-reported adverse mental health history and a partner's history of anxiety. Other studies have also reported an adverse mental health history as a risk factor for depression during this period (Nishimura and Ohashi, 2010; Wee *et al*., 2015). Adverse mental health whether recurring or newly developed in the early postnatal period has a deleterious impact on fathers, their parenting behaviours and their relationships (O'Brien *et al*., 2017). There is a correlation between paternal and maternal adverse mental health, which in turn escalates the risk of infant and child emotional and behavioural problems (Pilkington *et al*., 2015). In this study, a partner's history of anxiety was an associated factor for increased depressive symptoms. Couples who are experiencing adverse mental health are more likely to face conflict or distress in their relationship and less likely to be able to communicate the difficulties and experiences they are facing (Chhabra *et al*., 2020). Furthermore, when a mother experiences adverse mental health it can negatively impact on her ability to take care for her infant, which may add increased roles and responsibilities on fathers (Chhabra *et al*., 2020). Given the potential adverse consequences for a father and his family, there is a compelling argument for focusing on identifying and supporting fathers who are experiencing adverse mental health in the early postnatal period.

The early postnatal period is a pivotal time for fathers in terms of bonding with their infant and redefining their relationship with their partner. It is also often a challenging time for fathers (Glasser and Lerner-Geva, 2018); however, their mental health is generally overlooked, their needs are often unmet (Baldwin *et al*., 2019), and there are currently no specialised supports provided by the government in Ireland for fathers experiencing adverse mental health during the perinatal period (Health Service Executive, 2017). It is also during this critical period that fathers have contact with healthcare professionals (HCPs) such as midwives and doctors at the maternity hospital and public health nurses (PHNs) and general practitioners in the community. These contacts provide HCPs with a window of opportunity to identify fathers who are at risk of adverse mental health and to support them achieve optimum adaptation and well-being through increased awareness and discussions about their mental health (Huusko *et al*., 2018). Midwives, doctors, and PHNs due to their contact with fathers in the early postnatal are well positioned to incorporate paternal mental health information and education into existing antenatal classes and reinforce the information in the hospital after birth, during well baby check-ups and immunisations. However, at both the maternity hospital and in the community, fathers are not considered clients of the HCPs; therefore, midwives, PHNs, and doctors are potentially limited in the support that they can provide. Nevertheless, the Health Service Executive (HSE) in Ireland has adopted a health behaviour framework to ‘Make Every Contact Count’ between HCPs and those who engage with the health service in order to empower and provide support so that positive health outcomes can be achieved (HSE, 2017). By using this framework as a catalyst for action, contacts between HCPs and fathers during the early postnatal period could be used to increase awareness about the support resources that are available and to advise fathers about adverse mental health.

As fathers have highlighted the challenges that they experience trying to gain information and support while engaging with the maternity services (Hambidge *et al*., 2021), social media and online interventions could potentially serve as effective channels through which mental health awareness and supports could be promoted. Previous research has found that fathers seek health and support information online (Jaks *et al*., 2019). The benefits of online education may be especially critical for fathers during the early postnatal period as they attempt to balance the numerous demands placed upon them and have limited time to attend in person education and support sessions (Letourneau *et al*., 2011; Wynter *et al*., 2021). Furthermore, interactive web sites can help reduce the feelings of shame and stigma that men feel when seeking help from HCPs in traditional settings (Thorsteinsson *et al*., 2018).

## Future research

Paternal mental health during the perinatal period continues to be under-researched. Longitudinal studies are needed to build a more comprehensive picture of the paternal mental health throughout the perinatal period. Such studies will allow for a greater understanding of the variability and changes in mental health across the perinatal period. The existing research assessing paternal perinatal mental health has been predominately quantitative (Shorey and Chan, 2020). Fewer studies have explored men's experiences of their own perinatal mental health (Darwin *et al*., 2017). More qualitative research is needed to examine the views and experiences of first-time and subsequent fathers concerning their perinatal mental health, their perceptions of what makes perinatal mental health resources accessible and acceptable, the type of support fathers want, how this is provided, who provides it, and when would be the optimal time in the perinatal period to offer support. There is currently a paucity of research with diverse populations of fathers. Young fathers, gay fathers, unemployed fathers, non-resident fathers, stay at home fathers, fathers from ethnic diverse backgrounds, and lower socioeconomic groups are all under-represented in research on paternal perinatal mental health well-being (Baldwin *et al*., 2019).

## Limitations

In this study, the population was homogenous, with most fathers Irish, educated to third/university level, married, and in full-time employment. Therefore, the generalisability of the findings is limited. Another limitation is that a cross-sectional design was used. The cross-sectional design only identified associations. Longitudinal follow-up studies are necessary to identify causal inferences (Setia, 2016). Due to practical considerations for administering the instruments in a clinical setting, diagnostic measures were not used; therefore, the results were not objectively validated with a clinic interview. Data were collected at one site only and convenience sampling was used in the study which could potentially have led to the under-representation or over-representation of particular groups within the sample (Jager *et al*., 2017). In the current sample, the participants were predominantly married, employed, Irish, and had a university level education. These sample characteristics thus make it difficult to generalise the current study findings to men from more diverse backgrounds and minority groups. The manifestation of stress, anxiety, and depression symptoms might be different in a sample from more diverse and sociological backgrounds as the challenges they face might be different. In the multivariable analysis, forward stepwise regression was used to identify the best predictors of stress, anxiety, and depression symptoms. Limitations of this method of analysis have been identified (Tabachnick and Fidell, 2019); however, it was used in this study as it is an appropriate analysis method when there are many variables, and the researcher is interested in identifying a useful subset of associated factors (Thayer, 2002). Furthermore, the study had a very large number of independent variables (some of which were highly correlated), compared to a relatively small sample size; therefore, stepwise forward regression was an appropriate method for selecting the independent variables to be included in the model. Finally, 71% of the population in the study were educated to third/university level, which is a considerable portion of the population; however, as there was no breakdown of their qualification level i.e. PhD, masters, or degree, it was not possible to find out if there were differences within this large group.

## Conclusion

As the early postnatal period is filled with uncertainty and new challenges, it is often a challenging time for fathers. Understanding paternal mental health during this period is important, as it is a critical time for fathers as they develop their bond with their infant and redefine their relationships with their partner and wider society. This study builds on the previous body of literature related to paternal perinatal mental. A notable percentage of fathers experienced stress, anxiety, and depression symptoms which suggests that the early postnatal is a time of increased risk for adverse mental health. The findings from this study highlights that the current focus on depression does not accurately represent the substantive risk to paternal mental health wellbeing in the early postnatal. The study identified statistically significant factors associated with stress, anxiety, and depression including being a subsequent father, not living in a house, having a history of adverse mental health, and having a partner with a history of anxiety. If these results are replicated consistently in future research, they may assist HCPs identify and support fathers at risk of adverse mental health outcomes.
